# No evidence that a range of artificial monitoring cues influence online donations to charity in an MTurk sample

**DOI:** 10.1098/rsos.150710

**Published:** 2016-10-19

**Authors:** Timothy J. Saunders, Alex H. Taylor, Quentin D. Atkinson

**Affiliations:** School of Psychology, University of Auckland, Auckland, New Zealand

**Keywords:** prosociality, eye images, charity donation, reputation, online behaviour

## Abstract

Monitoring cues, such as an image of a face or pair of eyes, have been found to increase prosocial behaviour in several studies. However, other studies have found little or no support for this effect. Here, we examined whether monitoring cues affect online donations to charity while manipulating the emotion displayed, the number of watchers and the cue type. We also include as statistical controls a range of likely covariates of prosocial behaviour. Using the crowdsourcing Internet marketplace, Amazon Mechanical Turk (MTurk), 1535 participants completed our survey and were given the opportunity to donate to charity while being shown an image prime. None of the monitoring primes we tested had a significant effect on charitable giving. By contrast, the control variables of culture, age, sex and previous charity giving frequency did predict donations. This work supports the importance of cultural differences and enduring individual differences in prosocial behaviour and shows that a range of artificial monitoring cues do not reliably boost online charity donation on MTurk.

## 1. Introduction

Humans care deeply about their reputations [1]. If we know our choices will be made public, we act more prosocially [2–6]. Recent work has shown that simple but evolutionarily significant artificial monitoring cues, such as an image of a pair of eyes, can promote cooperation [7–22]. While an image alone cannot monitor behaviour, the evolutionary legacy hypothesis holds that humans possess an evolved proximate mechanism that causes us to react to monitoring cues as if our reputations are at stake [9]. Work using a range of economic games has shown that people act more prosocially when an image of eyes [7–13], or even simply three dots in the configuration of a face [14], is present in the environment. These effects have been replicated in real-world settings. In an early study, Bateson *et al.* [15] found people gave higher voluntary payments for coffee and tea when pictures of eyes, rather than flowers, were placed above an honesty box in a university tea room. Since this finding, images of eyes have also been shown to reduce littering [16–18], reduce bicycle theft [19], increase voter turnout [20] and even increase donations to charity [21,22].

However, several studies have failed to find evidence for a monitoring effect. Research using the trust and ultimatum economic games has found no effect of eyes on prosociality [23,24]. Dictator games played in total darkness [25], or after prolonged exposure to eye cues [26] have also found no effect of monitoring, unlike dictator games played under normal conditions, e.g. [10]. Other studies have found inconsistencies in the strength of the monitoring effect. Male eyes have been shown to increase prosociality more than female eyes in one study [15] but not in another [27]. Eyes that appear angry may have a greater prosocial effect [15]. Pictures of faces, rather than eyes, have not been shown to increase prosociality in a dictator game [7], yet a schematic of a face made of three monochrome dots does increase prosociality [14]. Finally, field studies have shown that the effect of eyes on prosociality is stronger when fewer people are in the environment [15,17,21,22]. These results, therefore, suggest that the effect of monitoring cues is to some extent dependent on the characteristics of the environment and the specific watching stimuli used.

Online testing, using sites such as Amazon Mechanical Turk (MTurk), allows the large-scale sampling of non-WEIRD populations [28,29]. Testing if the monitoring effect occurs in such an environment has the potential to create a number of applied benefits, such as reducing online antisocial behaviour or increasing online charity donation. However, work online has so far been inconclusive regarding the efficacy of the monitoring effect. Raihani & Bshary recently showed that images of eyes did not increase the amount of money shared by participants in an online setting [28], although subsequent re-analysis revealed eyes did increase the frequency of sharing itself [27].

Here, we examined whether a range of artificial monitoring cues can affect online charity donations across a large, culturally diverse sample of participants via MTurk. Rather than consider a single monitoring cue, we expanded the potential theoretical and applied implications of the study by considering a range of monitoring stimuli. Our aim was to test predictions derived from prior research and theory regarding the range of stimuli that activate our evolved reputational monitoring psychology. In addition, by considering a range of stimuli that varies along theoretically important dimensions, we maximize the chance of finding any monitoring effect in an online context. We considered the following range of monitoring stimuli:

1. *Eyes* versus *faces*: much of the research on artificial monitoring cues has focused on eye images. However, if these effects reflect an evolutionary legacy of selection for sensitivity to reliable predictors of actual monitoring, we would expect images of whole faces to be closer to the proper adaptive domain and so more effective than images of eyes. Conversely, it may be that the proximate mechanisms involved have evolved to indeed focus on eyes only, as the most important and salient cue for actual monitoring. Consistent with the latter, there is some suggestion from prior research that faces are less effective than eye images alone [7], though this study varied several features of the images, including the number and orientation of faces versus eyes. Here, we used a set of comparable face and eye images to directly compare the efficacy of eye versus face effects.
2. *Abstract representation* versus *photograph*: Previous work has found a monitoring effect extends to abstract monitoring cues [7,9–11,13,14,22,23,25,26,28], including a minimal face cue comprised of three dots oriented in an inverted pyramid [14]. This suggests that a monitoring cue effect ‘might be so fully ingrained into unconscious social cognition that it could be set off by a weak stimulus’ [14]. However, the effect of abstract relative to photo-realistic images has never been tested. Here, we included an abstract face image and an inverted control version of this image, both taken directly from Rigdon *et al.* [14].
3. *Emotion*: It remains unclear whether any sensitivity to eye or face cues is influenced by the emotion expressed in the stimuli. To the extent that monitoring cue effects reflect an evolutionary legacy of selection on sensitivity to fitness-relevant monitoring stimuli, we might predict that eyes or faces that express anger would be more threatening, entailing potentially greater fitness consequences, and so generate a larger monitoring cue effect. Previous findings have suggested that emotion may influence the efficacy of the monitoring cues [15], but this has not been rigorously tested. Here we compared stimuli showing angry faces with both sad (negatively valenced but non-threatening) and neutral faces.
4. *Number of eyes/faces*: The number of observers of a prosocial behaviour is likely to have been important for reputation management as the greater the number of observers, the greater the chance of a behaviour being observed and reported to others. Another prediction based on the evolutionary legacy hypothesis is, therefore, that stimuli that depict a greater number of monitors may have a greater effect on behaviour. To test for this effect, we varied the number of faces or eyes presented across stimuli.

To quantify and control for the effect of stable individual differences in prosociality, we also considered a broad range of possible covariates including age, sex, education, past prosocial behaviour, personality, and religious, cultural and political differences.

## 2. Material and methods

### 2.1. Participants

Participants were sampled from the crowdsourcing Internet marketplace, MTurk (www.mturk.com). After screening (see Measures and procedure for details), 1535 people were randomly allocated to this study (879 male, 656 female; *M*_age_ = 31.5, s.d._age_ = 10.3; 65% White, 22% Asian; 80% from USA, 16% from India). Data collection was carried out from August 2013 to December 2014 under the ethics approval of the University of Auckland (reference no. 8933). All research methods were approved by the University of Auckland Human Participants Ethics Committee. Participants were told that ‘by completing the questions in our survey you are consenting to being a part of this study’ at the start of the survey. Completed surveys, therefore, provided written consent for participation in our study. Participants were allocated to conditions using random assignment functions in SurveyMonkey (www.surveymonkey.com). All participants were paid US$0.20 for their participation and had the opportunity to keep a further US$0.80.

### 2.2. Measures and procedure

MTurk was used as a means to advertise the study and to recruit a large number of participants. The MTurk marketplace has been shown to be a useful and reliable population for behavioural researchers to collect large samples of people from diverse backgrounds [30]. MTurk linked marketplace workers to an online survey created using SurveyMonkey. The first measures in the survey asked participants basic demographic and screening questions before they were linked to the experimental part of the survey. The aim of screening workers was to avoid responses from restricted populations [30], people who went quickly through the survey without reading the questions, and people with poor English ability. The screening task was a simple reading comprehension task in which MTurk workers were required to answer two simple multi-choice questions where the answer could be attained from the accompanying passage of text (see [31], for an example). This simply required English proficiency and taking time to read the passage before choosing an answer. Approximately 63% of workers answered both these questions correctly and were, therefore, linked to the study. The experimental part of the survey included the prime images and the dependent measure, followed by the covariate measures.

The study was advertised on MTurk as an ‘academic survey on demographics and decision making’. To qualify workers had to have completed 100 approved hits or more, with a hit approval rate greater than or equal to 98%. It took approximately 10 min to complete and paid US$0.20 with the chance to get a US$0.80 bonus. Workers who chose to take the survey were linked to SurveyMonkey and to the first stage of the survey which consisted of a briefing page, some demographic questions and the screening task. Participants were told that all their answers would remain confidential and they were asked to give consent to being part of the study. The demographic questions were completed and then the two screening questions. The participants who did not answer both screening questions correctly were linked to an end of survey page where they were thanked for their time. The participants who passed the screening were linked to the priming page. The random assignment function in SurveyMonkey randomly produced one of the primes for each participant. Participants were told that they had earned a bonus of $0.80 and that they were free to keep it all for themselves, or they could choose to donate any proportion of it to the charity UNICEF (The United Nations Children's Fund). Below the image, participants were required to type in both how much they wanted to keep and how much they wanted to donate to UNICEF. Participants were then asked to complete a measure of their mood on the next page after they made their decision. The rest of the covariate items were asked and then participants were thanked for their time.

There were 34 different prime images used in total, representing 11 conditions (table 1). In six of the experimental image conditions, we used pseudo-replicates of multiple different images to minimize the probability that our findings are due to our specific image choices. To examine the effect of emotion, we included neutral face, angry face and sad face conditions. To examine the effect of the number of observers, we included a group of three neutral faces. To determine the relative efficacy of eyes versus faces we included a ‘neutral eyes’ and a ‘group of three pairs of neutral eyes’ condition. We also included a schematic neutral face (following Rigdon *et al.* [14]), plus schematic angry face and a schematic sad face, as well as a schematic inverted face (control) and two blank controls (one black and one white). Eighteen images were sourced from the Cohn–Kanade AU-Coded Facial Expression Database v. 2 (CK+) which are fully FACS (Facial Action Coding System) coded and have validated emotion labels [32,33]. Only a limited number of images from the database had validated emotion labels for all three facial expressions we wanted to test (neutral, angry and sad). From these, three males and three females were selected, each with an image for all three facial expressions. The following subjects from the CK+ database were used in this study: S066, S113, S130, S501, S504 and S506. Two group images were created from the same selected images using the neutral faces arranged in a random order. The three females and the three males were merged side by side using Adobe Photoshop CS5.1 to create two new images. The backgrounds were blended to make it appear as though the images were taken from the same photograph. To create the eyes-only and group eyes-only images, the neutral face images and group images were simply cropped to show only the eyes. The schematic face and inverted schematic face images [14] were made using three black dots against a white background. Two additional schematic images were created by adding slanted (25° from the horizontal) rectangular ‘eyebrows’ above the eyes to depict either anger or sadness. The final two images were control images of a white square on a black background and a black square on a white background. The prime images were all displayed in greyscale and were approximately 300 × 300 pixels in size with the exception of the eyes-only neutral images which were approximately 300 × 50, and group images which were approximately 1000 × 300 for the group faces and 1000 × 50 for the group eyes. The size of the eyes between face, groups and eyes-only images was held constant.

**Table 1.** Characteristics of each condition.

prime condition	photo or schematic	eyes or face	emotion	individual or group	pseudo-replicates
control	—	—	—	—	2
neutral face	photo	face	neutral	individual	6 (3 male, 3 female)
angry face	photo	face	angry	individual	6 (3 male, 3 female)
sad face	photo	face	sad	individual	6 (3 male, 3 female)
group faces	photo	face	neutral	group	2 (1 male group, 1 female group)
neutral eyes-only	photo	eyes	neutral	individual	6 (3 male, 3 female)
group eyes-only	photo	eyes	neutral	group	2 (1 male group, 1 female group)
schematic face	schematic	face	neutral	individual	1
schematic inverted	—	—	—	—	1
schematic angry	schematic	face	angry	individual	1
schematic sad	schematic	face	sad	individual	1

We recruited 1535 participants across these 11 conditions in order to maximize our power to detect an effect of each prime on donation behaviour. Our sample sizes for each condition ranged between 100 and 179. Previous work examining the effect of priming eyes on prosocial giving has found effect sizes of between 0.30 and 0.59 [10,13]. More recently, Raihani & Bshary [28] did not find an effect of eye images, but did find a small but significant effect for an image of flowers, with an effect size of between 0.25 and 0.71. Based on an average effect size of 0.45, our power to detect a pairwise effect between our control condition and any one experimental condition at *α* = 0.05 is 0.965–0.991.

In addition to donation responses, participants completed survey items measuring a range of demographic and individual differences potentially associated with prosociality. As part of the screening survey, we collected basic demographic data on participant age, sex and education (*less than high school degree* (1), *high school degree or equivalent* (2), *some college but no degree* (3), *associate degree* (4), *bachelor degree* (5), *or graduate degree* (6)). We included three measures of ethnic background—stated ethnicity (*Asian, African American, White, Hispanic or Latino, other*), first language (grouped by the six most common languages plus a category for ‘other’ languages) and country of origin. To compare our findings with Raihani & Bshary [28], country of origin was categorized into the same 10 categories classified in [34]. This categorized each country into one of nine world cultures along with one category for countries not defined. We also asked participants about a range of potential covariates of prosocial giving, including items relating to mood, religion, values and past behaviour. These questions were asked after the prime and donation opportunity so they did not interfere with the effects of the prime.

Mood has been shown to influence prosociality (e.g. [35]). We used a shortened version of the Positive and Negative Affect Schedule to measure the participant's mood at the time of the study [36]. Participants were required to rate on a 5-point scale from *not at all* (1) to *very* (5) to what extent they felt each one of 10 emotions (e.g. upset). This gave a measure of negative affect (Cronbach's *α* = 0.83) and positive affect (Cronbach's *α* = 0.83).

A growing body of research suggests that religiosity and religious belief may promote prosocial behaviour [37,38]. In order to account for religious variation in our sample, participants were asked to state their religion. We also included nine other religiosity measures. Three items, from Gervais & Norenzayan [39], ask for level of agreement with the statements ‘God exists’, ‘The devil exists’ and ‘Angels exist’. Another item, ‘It is likely that God, or some other type of spiritual non-human entity, controls the events in the world’ was taken from Laurin *et al.* [40]. Another three items were adapted from these existing items. The adapted items we created were ‘God, or some other supreme being, is watching over me’, ‘God, or some other supreme being, punishes those who do wrong’ and ‘Religious leaders communicate the will of God, or the will of some other supreme being’. Participants were required to rate their agreement with each item on a 7-point scale ranging from *strongly disagree* (1) to *strongly agree* (7). Participants were also asked to report religious attendance using the item ‘How often do you take part in religious services e.g. attend church?’. This was rated on a 6-point scale ranging from *never* (1) to *once or more a week* (6). All the above items had a high correlation (>0.65) with the last item, ‘How religious do you consider yourself?’ which was rated on a 5-point scale from *not religious at all* (1) to *very religious* (5). These nine items (including the item ‘How religious do you consider yourself?’) were, therefore, combined to produce a religiosity score for participants. Internal consistency of this religiosity measure was maximized when all nine items were included (Cronbach's *α* = 0.96). In addition to the nine religiosity items, we asked a single item question, ‘There is no such thing as karma’, to examine whether a belief in karma [41], either an Eastern interpretation or a Western interpretation (not necessarily tied to any religion or deity), affected donation levels.

Political views may affect prosociality via attitudes towards income inequality and the moral obligation to help those in need [42]. Thus, we measured left–right political orientation using a 5-item scale (Cronbach's *α* = 0.82) and one probe item for libertarian–authoritarian orientation, ‘Schools should teach children to obey authority’ [43]. Participants were required to rate their agreement with each item on a 7-point scale ranging from *strongly disagree* (1) to *strongly agree* (7). We also included two items designed to measure faith in secular institutions of social justice (‘The Government will look after me if I get sick’ and ‘The police make my world safe’).

In order to measure the extent to which participants cared about their reputation and hence their sensitivity to monitoring cues, we included measures of antisociality and public self-consciousness. Anti-social attitudes were measured using items from the World Values Survey [44]. Participants were asked to rate how justifiable were moral transgressions such as ‘Avoiding a fare on public transport’. Each item was rated on a 10-point scale ranging from *never justifiable* (1) to *always justifiable* (10). Public self-consciousness was measured using an existing 7-item measure [45] (e.g. ‘I usually worry about making a good impression’) and each item was rated on a 5-point scale from *not at all like me* (1) to *very much like me* (5).

Finally, past behaviour is expected to be a good predictor of prosociality [46]. In order to examine whether self-reported past charitable behaviour predicts donation levels, participants were asked how many hours they typically volunteer each month, and how many times they donated to charity in the last 12 months.

### 2.3. Design and data analysis

We used a between-subjects design. The dependent measures were the amount that participants chose to donate to the charity UNICEF when they were allocated a bonus US$0.80, and the probability of donating something rather than nothing. The independent variable was the type of image prime shown on screen while the participant made this decision. Images were combined into their associated conditions (control, neutral face, angry face, sad face, group faces, neutral eyes-only, group eyes-only, schematic face, schematic inverted, schematic angry and schematic sad). The R v. 3.0.1 statistical package (www.r-project.org) was used to perform all statistical tests. Data screening revealed a highly zero-inflated distribution for donation choice (52% of participants did not donate anything). This distribution was in line with previous work by Raihani & Bshary [28] using the dictator game paradigm on MTurk. The data could not be transformed for parametric testing so non-parametric testing was used for analysing differences in amount donated. Probability of donating between conditions was analysed using *χ*^2^-tests. Following Raihani & Bshary [28], we used ordered logistic regression as a technique to include covariates in our analysis. Eight ordered categories for amount donated were created based on the most popular allocations. For our model selection process, because we had a high number of independent variables, we only considered variables that showed a significant relationship with our new ordinal donation variable based on bivariate comparisons. We then generated a set of models of all possible combinations of these variables and inferred the best models based on corrected Akaike information criterion [47–49]. We also calculated the probability of each variable being included in the best model using Akaike weights (for a detailed description and example of this method, see [48]). The full dataset for this study is available from http://figshare.com/articles/Data_S1/1032615.

## 3. Results

The probability of making a donation and the mean value donated for each of the 11 prime image conditions is shown, together with standard deviation, in table 2. A Kruskal–Wallis analysis of variance (ANOVA), adjusted for ties, was performed to test for differences in amount donated between the conditions. This revealed that there was a significant difference in the amount donated between conditions (*H*(10) = 18.8, *p* = 0.04); however, post hoc testing (pairwise comparisons of the mean ranks [50]) revealed no significant pairwise differences (*p* > 0.05). A *χ*^2^-test also revealed no relationship between condition and whether a donation was made (*χ*^2^ (10, *N* = 1535) = 11.7, *p* = 0.31). For completeness, we combined all monitoring conditions (neutral face, angry face, sad face, schematic neutral face, schematic angry face, schematic sad face, group of three neutral faces, neutral eyes and group of three neutral eyes) and compared this new ‘monitoring’ condition with the combined control conditions (schematic inverted face and black and white square controls). We found no significant difference between the monitoring (M = $0.14, Mdn = $0) and control (M = $0.14, Mdn = $0) conditions in amount donated (Mann–Whitney test: *U* = 156561.5, *p* = 0.8). The probability a donation was made in the monitoring condition was 48% and the probability a donation was made in the control condition was also 48%. We found no significant difference between monitoring and control in the probability of donating (*χ*^2^ (1, *N* = 1535) < 0.001, *p* = 0.99).

**Table 2.** Probability of making a donation and mean donation in US$ for each prime condition with s.d. and sample size.

prime condition	Probability donation > 0	mean	s.d.	*N*
control	0.48	0.15	0.22	145
neutral face	0.52	0.17	0.23	168
angry face	0.51	0.15	0.2	179
sad face	0.54	0.18	0.23	177
group faces	0.45	0.15	0.22	122
neutral eyes-only	0.53	0.16	0.23	176
group eyes-only	0.43	0.12	0.19	128
schematic face	0.44	0.12	0.18	110
schematic inverted	0.49	0.14	0.2	100
schematic angry	0.42	0.08	0.12	105
schematic sad	0.43	0.12	0.19	125

With no significant main effects of image primes, the effects of possible covariates were explored. Table 3 shows bivariate analyses of our full set of candidate covariates. Of the 19 covariates, we found that there was a significant effect for 13. The table reveals a significant difference between male and female participants in the amount they donated. Females (M = $0.17, Mdn = $0.05) donated more than males (M = $0.13, Mdn = $0). There were also significant differences in amount donated between world cultures. Pairwise comparisons of the mean ranks [50] indicated that the only significant difference was between English-speaking and South Asian cultures. While none of the other cultural regions showed significant differences, the sample sizes were small (less than 20) because the bulk of the MTurk population is from USA and India. Participants classified as coming from a South Asian culture, based on country of origin [34], donated significantly more (M = $0.23, Mdn = $0.2) than participants classified as coming from an English-speaking culture (M = $0.13, Mdn = $0) (*difference (in mean ranks)* = 103.4, *p* < 0.05). Correspondingly, there was a significant difference in amount donated between ethnicity and reported first language groups. Asian participants donated significantly more (M = $0.19, Mdn = $0.1) than White participants (M = $0.13, Mdn = $0) (*difference* = 77.9, *p* < 0.05) and participants who reported that their first language was Tamil donated significantly more (M = $0.27, Mdn = $0.3) than participants who reported that their first language was English (M = $0.13, Mdn = $0) (*difference* = 166.4, *p* < 0.05). There were also significant differences between first languages English (M = $0.13, Mdn = $0) and Malayalam (M = $0.22, Mdn = $0.2) (*difference* = 211.2, *p* < 0.05) and other (M = $0.18, Mdn = $0.1) and Tamil (M = $0.27, Mdn = $0.3) (*difference* = 217.1, *p* < 0.05). Table 3 also shows significant group differences between religions (Kruskal–Wallis test, *H*(9) = 76.6, *p* < 0.001). Participants who reported having no religion donated significantly less (M = $0.12, Mdn = $0) than participants who reported being Christian (M = $0.15, Mdn = $0.05) (*difference* = 82.3, *p* < 0.05) as well as participants who reported being Hindu (M = $0.22, Mdn = $0.2) (*difference* = 123.8, *p* < 0.05). The difference between Christian and Hindu was also significant in that participants who reported being Hindu donated more than participants who reported being Christian (*difference* = 126, *p* < 0.05). There was a significant difference in the amount donated across different qualifications; however, post hoc testing (pairwise comparisons of the mean ranks [50]) revealed no significant pairwise differences (*p* > 0.05). Table 3 also shows that amount donated was positively correlated with age, positive affect, religiosity, volunteering frequency, charity giving frequency and authoritarianism, and negatively correlated with antisociality.

**Table 3.** Bivariate relationships between each predictor variable and donation amount.

	donation amount (ordered categories)		
predictor	statistical test	test statistic	*p*-value
sex (male)	Mann–Whitney *U*	*U* = 318953.5	<0.001
qualification	Kruskal–Wallis	*H* = 11.8	0.04
culture	Kruskal–Wallis	*H* = 104.6	<0.001
language	Kruskal–Wallis	*H* = 77.4	<0.001
ethnicity	Kruskal–Wallis	*H* = 45.2	<0.001
religion	Kruskal–Wallis	*H* = 76.6	<0.001
age	Spearman's *ρ*	*ρ* = 0.09	<0.001
positive affect	Spearman's *ρ*	*ρ* = 0.14	<0.001
negative affect	Spearman's *ρ*	*ρ* = 0.007	0.79
karma	Spearman's *ρ*	*ρ* = 0.03	0.19
religiosity	Spearman's *ρ*	*ρ* = 0.16	<0.001
volunteering frequency	Spearman's *ρ*	*ρ* = 0.16	<0.001
charity giving frequency	Spearman's *ρ*	*ρ* = 0.22	<0.001
government control	Spearman's *ρ*	*ρ* = 0.005	0.84
police control	Spearman's *ρ*	*ρ* = 0.003	0.9
left wing	Spearman's *ρ*	*ρ* = 0.033	0.19
authoritarianism	Spearman's *ρ*	*ρ* = 0.14	<0.001
antisociality	Spearman's *ρ*	*ρ* = −0.1	<0.001
public self-consciousness	Spearman's *ρ*	*ρ* = 0.025	0.32

As the above covariates are potentially confounded, we used binary logistic regression to identify the strongest independent predictors of amount donated. We considered all of those predictors in table 3 that showed a significant bivariate relationship with amount donated, plus the prime variable. None of the variables in table 3 showed a significant interaction with prime in a bivariate analysis using ordered logistic regression so our model selection process did not include interaction effects. We analysed 16 384 models comprising all possible combinations of the 14 variables using ordered logistic regression. Table 4 shows relative variable importance as the probability (calculated from Akaike weights, see [48]) that the best model includes each variable. Table 4 shows clear support for models including charity giving frequency, sex, culture and age, all of which have a relative variable importance of greater than 90%. A model including sex, age, culture and charity giving frequency was also identified as the best model from our candidate set with the fewest predictors. Table 5 summarizes results for a model predicting donation amount from these four predictors only. This confirms significant independent effects of each predictor. Age and charity giving frequency are positively correlated with donation amount, while being male is negatively correlated with donation amount. The culture effect is largely driven by significantly higher donation amounts in the South Asian, Latin American and other cultural areas, relative to participants from the English-speaking cultures.

**Table 4.** Probability of each variable being included in the best model predicting amount donated.

variable	probability that the best model includes the variable
charity giving frequency	>0.99
sex	0.99
culture	0.99
age	0.91
antisociality	0.52
authoritarianism	0.51
volunteering frequency	0.38
religiosity	0.32
positive affect	0.28
qualification	0.27
ethnicity	0.21
first language	0.13
religion	0.01
prime grouped	0.006

**Table 5.** Model summary showing parameter estimates from a model predicting donation amount from sex, age, charity giving frequency and culture (*n* = 1535).

predictor	*β* (s.e.)	odds ratio	*p*-value
*intercepts*			
1|2	1.247 (0.201)		
2|3	1.467 (0.202)		
3|4	1.915 (0.204)		
4|5	2.307 (0.207)		
5|6	2.711 (0.210)		
6|7	4.034 (0.229)		
7|8	4.401 (0.239)		
sex: male (*n* = 879)	−0.374 (0.101)	0.688	<0.001
age	0.014 (0.005)	1.014	0.003
charity giving freq.	0.219 (0.032)	1.245	<0.001
*culture*			
Africa (*n* = 2)	0.404 (1.232)	1.498	0.743
Catholic Europe (*n* = 11)	0.307 (0.603)	1.358	0.611
confucian (*n* = 6)	0.069 (0.865)	1.072	0.936
English speaking (*n* = 1244)	—	—	—
ex-communist (*n* = 2)	0.643 (1.011)	1.902	0.525
Latin America (*n* = 6)	1.476 (0.676)	4.375	0.029
orthodox (*n* = 11)	0.842 (0.567)	2.321	0.138
other (*n* = 15)	1.448 (0.426)	4.253	< 0.001
Protestant Europe (*n* = 6)	1.190 (0.765)	3.286	0.120
South Asia (*n* = 232)	1.117 (0.128)	3.057	<0.001

## 4. Discussion

We found no evidence that MTurk participants in our online survey donated more to charity or were more likely to give to charity in the presence of a diverse range of artificial monitoring stimuli. Priming faces or eyes did not affect donation behaviour, regardless of the real or abstract nature of the images used, the number of eyes or faces or the emotion expressed. These stimuli include images of eyes almost identical to those used in past studies (e.g. [8,14–21,27,28]) and images of faces or the same face schematic previously shown to increase prosociality [14]. These findings do not allow us to test theoretical predictions regarding the relative efficacy of the different monitoring cues because none of the monitoring cues produced a significant effect on donation behaviour.

We did, however, find that sex, broad cultural background, age and past donation frequency influenced the amount donated. Females were found to donate more than males, replicating Nettle *et al.*'s [27] findings. Consistent with previous work [28,51,52], we found large differences in prosocial giving across widely recognized cultural divisions [34]—in particular, English-speaking versus South Asian cultures. Donation also increased with age and frequency of past charity donations, as in past research [28,46,53,54]. Thus, though we did not detect a monitoring effect, we did replicate a number of other recent findings regarding the predictors of human prosociality.

In their recent study, Raihani & Bshary [28] found that flowers had a stronger effect on the amount donated than eyes when subjects participated in an online dictator game. Subsequent reanalysis by Nettle *et al.* [27] showed that eyes did affect the probability of a donation occurring, but not the amount donated. It is not clear why we did not replicate either the effect of eye images on probability of donation seen in Raihani and Bshary or more general monitoring effects using any of our 31 treatment images.

One simple explanation for our results is that any potential effects of artificial monitoring cues are so weak as to not appear in our study despite our large sample size. Another possibility is that the cues we used may have varied in important ways from those used in previous studies. However, we think this is highly unlikely given that the majority of our images were almost identical to those used in previous studies where an effect was found, and some were actually identical.

A third potential explanation concerns the amount of attention received by the cues in our study. Recent work has shown that if cues are dwelt on too long they cease to have efficacy [26]. It is possible our cues, presented on the donation page itself, were somehow too obvious, in that participants paid them too much attention, and thus a monitoring effect did not emerge. However, given that our methodology was highly similar to studies where positive effects have been found this seems unlikely.

A fourth explanation is the nature of the online environment and participant pool. Raihani & Bshary's [28] study, which was run online and recruited participants from MTurk also found weak or negative monitoring effects (an image of flowers was associated with increased donation amount compared with an image of eyes). Our findings are consistent with Raihani & Bshary's [28] hypothesis that the effect of monitoring cues may be weakened or non-existent in an online environment. Raihani and Bshary suggest this may be due to a cloak of perceived anonymity when online that may render participants impervious to the effect of monitoring cues. Conversely, it may be that the perception of monitoring is in fact greater online, particularly among those recruited via MTurk, because participants may feel their performance on the task is being monitored and could affect their pay-offs. If all participants already feel they are being monitored, this may override any effect of monitoring cues in the treatment conditions.

There are also other features of MTurk samples that may explain our null findings. Participants recruited through MTurk have considerable experience participating in research [55]. This may encourage automatic responding. However, our respondent filtering mechanism minimizes the probability of automatic responding and the observed effects of sex, cultural background, age and past donation frequency indicates that our MTurk sample was producing response patterns in line with other online and in-person dictator game studies. Some have speculated that experienced participants will also be experienced at ignoring stimuli that are not relevant to the task [26] and so would not be influenced by task irrelevant images on the screen. It is also possible that our screening task inadvertently selected for individuals who were more cooperative or less concerned about their reputation. While, we cannot rule out the possibility that our findings based on participants recruited through MTurk are biased in some way, we note that these issues would also apply to studies which did find evidence of the monitoring effect using students and paid participants, e.g. [10,13], yet used subjects recruited via either University participant pools (where participants are highly experienced and uniquely well-versed in many of the theories being tested) or other forms of paid participation (which can bias participation and create experimenter demand effects). All of the above concerns also need to be weighed up against the advantages of an MTurk sample—in particular, studying behaviour beyond the normal Western undergraduate sample used in psychology. It is also worth noting that, while we found no monitoring cue effects, a re-analysis of Raihani and Bshary's MTurk study did find a positive effect of eyes on the probability of giving [27]. If this effect is robust, then an MTurk sample has generated monitoring effects previously, suggesting that more research is needed into the factors affecting monitoring cues in online studies like those using MTurk.

Our findings add to the growing body of evidence that artificial monitoring effects do not influence human prosociality in a uniform way [7,10,21–27,56]. In our study, we tested for an effect of monitoring cues across a total of 11 conditions, including multiple pseudo-replicates across six experimental image conditions. Given this range of monitoring stimuli, our large sample sizes and our replication of past studies showing that sex [27], culture [28,51,52], age [28,53,54] and past donation frequency [46] influence the amount donated, our study indicates that artificial monitoring cues are not a strong or reliable booster of online charity donations on MTurk. As discussed above, it is not yet clear why this is the case. Further research in this area has the potential to not only uncover the cognitive underpinning of the artificial monitoring effect, but also allow the development of novel techniques to promote prosocial behaviours in online environments.

## Data accessibility

The full dataset for this study is available from http://figshare.com/articles/Data_S1/1032615.
